# P-46. A Comparative Analysis of Vaccine Uptake in Face-to-Face vs. Virtual Care for Active Duty Air Force Members Living with HIV

**DOI:** 10.1093/ofid/ofae631.253

**Published:** 2025-01-29

**Authors:** Paul J Wurtz, Laquonda Finney, Joseph Yabes

**Affiliations:** Brooke Army Medical Center, Converse, Texas; Brooke Army Medical Center, Converse, Texas; Brooke Army Medical Center, Converse, Texas

## Abstract

**Background:**

Military regulation requires US Air Force (USAF) members living with HIV receive Infectious Disease evaluation at the Medical Evaluation Unit (MEU). With a worldwide distribution of the USAF, telehealth utilization began in 2020 with improvements in clinical outcomes and decreased costs. However, not all facets of care are easily amenable to telehealth. We aimed to review differences in vaccination rates since transition from face to face (F) to virtual (V) visits.

**Figure:**
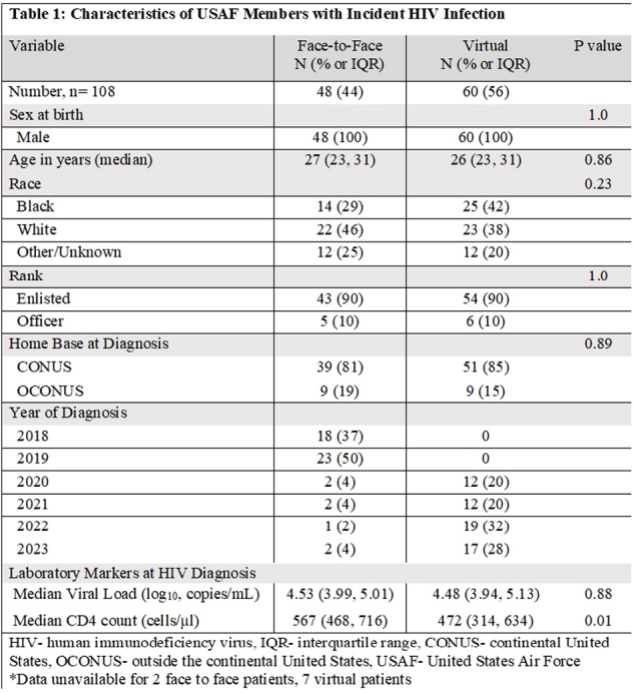

**Methods:**

All USAF active-duty members with incident HIV diagnosis between 2018 and 2023 were included. Only airmen stationed local to the MEU were evaluated F after 2020. Demographic, and clinical information was compiled. Vaccines of interest were selected according to current guidelines: human papilloma virus (HPV), meningococcal ACWY, pneumococcal, recombinant zoster and Mpox vaccines. Vaccine completion rates and time from HIV diagnosis to first vaccine dose was compared between groups.

**Figure:**
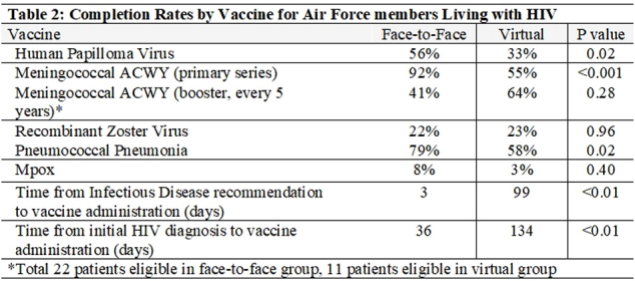

**Results:**

All 108 patients were male. Forty-four percent of visits occurred F and 56% were V. Median age was similar, 27 years for F and 26 years for V (p=0.84). Overall, demographics were not significantly different between groups (Table 1). Initial CD4 count was lower among those who received V care (472 vs 567; p=0.01). It took longer for patients to receive their first vaccine dose with V visits compared to F visits (99 vs 3 days; p< 0.01) (Table 2). Likewise, days from HIV diagnosis to first dose of vaccine administration was longer with V visits compared to F (134 vs 36; p< 0.01). Vaccine completion rates varied by type. Pneumococcal and HPV completion rates were higher in the F group compared with V group (p=0.02). Completion rates of the meningococcal ACWY primary series were similarly higher in the V group 92% vs 55%; p=< 0.001). Mpox completion rates were higher with F visits but not statistically significant (8% vs 3%; p=0.4). Recombinant zoster vaccine completion rates were nearly identical (22% vs 23%; p=0.96).

**Conclusion:**

Vaccination rates were lower and took longer among USAF personnel who underwent V visits compared to F. As healthcare systems increasingly employ telehealth, future studies should investigate provider and patient attitudes on vaccine counseling during V visits to ensure the delivery of high-quality care in the virtual era.

**Disclosures:**

**All Authors**: No reported disclosures

